# Diagnosis of hypoprolactinemia

**DOI:** 10.1007/s11154-024-09896-8

**Published:** 2024-07-22

**Authors:** Emre Urhan, Zuleyha Karaca

**Affiliations:** Department of Endocrinology, Erciyes University Medical School, Kayseri, Turkey

**Keywords:** Prolactin, Hypoprolactinemia, Hypopituitarism, Low prolactin, TRH stimulation test

## Abstract

Prolactin is a polypeptide hormone composed of 199 amino acids, synthesized by lactotroph cells. Its primary effects are on the mammary gland and gonadal axes, but it also influences different organs and systems, particularly metabolic functions. Current literature has mainly focused on the diagnosis, monitoring, and treatment of hyperprolactinemia. Due to the lack of a well-established effective treatment for hypoprolactinemia, it is not clinically emphasized. Therefore, data on its diagnosis is limited. Hypoprolactinemia has been associated with metabolic dysfunctions such as type 2 diabetes mellitus, fatty liver, dyslipidemia, fertility problems, sexual dysfunction, and increased cardiovascular disease. While often seen as a part of combined hormone deficiencies due to pituitary damage, isolated prolactin deficiency is rare. Hypoprolactinemia can serve as a marker for extensive pituitary gland damage and dysfunction.

Low or undetectable serum prolactin levels and the absence of a sufficient prolactin peak in the thyrotropin-releasing hormone (TRH) stimulation test are considered diagnostic for hypoprolactinemia. Gender appears to influence both basal prolactin levels and TRH stimulation test responses. Basal prolactin levels of, at least, 5 ng/mL for males and 7 ng/mL for females can be used as cut-off levels for normal prolactin reserve. Minimum peak prolactin responses of 18 ng/mL for males and 41 ng/mL for females to TRH stimulation can exclude hypoprolactinemia. However, larger population studies across different age groups and sexes are needed to better define normal basal prolactin levels and prolactin responses to the TRH stimulation test.

## Introduction

The data on diagnostic approaches to hypoprolactinemia is limited since hypoprolactinemia is not considered to be important, furthermore it is usually neglected in both the definition and diagnosis of hypopituitarism [1]. However, awareness of the clinical significance of low prolactin levels has increased recently and growing attention is paid to the diagnosis of hypoprolactinemia. In this review, we have addressed and examined the diagnosis of hypoprolactinemia.

Prolactin is a polypeptide hormone composed of 199 amino acids, synthesized by lactotroph cells that constitute 15–20% of the anterior pituitary [2]. Lactotrophs are the major source of circulating prolactin. Additionally, other tissues such as adipose tissue, lymphoid cells, mammary epithelial cells, prostate, sweat glands, and skin fibroblasts also contribute to prolactin secretion, though to a lesser extent [3].

To date, the presence of a prolactin-releasing hormone has not been identified, and the fundamental regulatory mechanism of prolactin synthesis and secretion is dopamine inhibition. Tuberoinfundibular dopaminergic neurons of the arcuate nucleus, which secrete into the median eminence, are hypothalamic neurons required for prolactin regulation through the portal blood. Simultaneously, prolactin also regulates dopamine release [4–7]. Stimulatory factors such as thyrotropin-releasing hormone (TRH), vasoactive intestinal peptide, oxytocin, ghrelin, estrogen, fibroblast and epidermal growth factors, vasopressin, and opioids are able to increase prolactin levels [8]. Factors such as histamine, somatostatin, norepinephrine, and serotonin have inhibitory effects on prolactin secretion [5].

The main effect of prolactin is on the mammary gland where it promotes the growth and development of the gland, maintain the production and continuity of breast milk via prolactin receptor (prolactin-R) [9]. The effects of prolactin is not limited with lactation, other effects on different organs and systems such as behavior, neurogenesis, neuroprotection, regulation of immune response, fluid-electrolyte balance, and homoestasis have been shown [9]. Prolactin affects follicle stimulating hormone (FSH) and luteinizing hormone (LH) levels through gonadotropin hormone-releasing hormone (GnRH), thus influencing testicular and ovarian functions. Prolactin-R is present in the testes, particularly on Leydig, Sertoli, and germ cells, influencing FSH and LH receptor expression and regulating spermatogenesis, playing a role in male fertility similar to its role in females [8].

Prolactin enhances adipogenesis and insulin sensitivity in adipose tissue and inhibits lipolysis. It plays a role in the metabolic functions of the liver by increasing insulin sensitivity and reducing fat accumulation, thereby preventing fatty liver [10]. The decreased expression of prolactin-R in the liver has been shown to lead to insulin resistance throughout the body [11]. Furthermore, increased prolactin-R expression in the hypothalamus was shown to increase insulin sensitivity [12]. Prolactin promotes beta-cell proliferation besides decreasing its apoptosis and enhances glucose-induced insulin secretion in the pancreas [10]. It has been shown that prolactin-R deficiency in the pancreas leads to beta-cell hypoplasia and impaired glucose-stimulated insulin response, resulting in glucose intolerance [13]. The relationship between prolactin and glycemic status has also been investigated in animal models. In streptozotocin-induced diabetic or diet-induced obese rats, prolactin treatment improved metabolic profile [14, 15], and these rats were shown to have more severe metabolic dysfunction when they lacked prolactin-R [14]. Additionally, pregnant mice lacking prolactin-R in their pancreas developed gestational diabetes [16].

Prolactin levels show variations in physiological and pathological changes of metabolism [10]. Low and higher prolactin levels lead to negative metabolic consequences [17, 18].

Hypoprolactinemia is commonly observed as a combined deficiency with other anterior pituitary hormone deficiencies. However, in very rare cases, isolated prolactin deficiency can also occur, and there have been a few case reports in the current literature [1, 19].

The most common cause of isolated hypoprolactinemia is treatment with dopamine agonists (DAs). Sheehan syndrome (SS), pituitary apoplexy, radiotherapy are leading causes of hypoprolactinemia, but traumatic brain injury (TBI), pituitary adenoma, autoimmunity, infiltrative and inflammatory diseases of the pituitary gland, empty sella, and genetic mutations can also lead to hypoprolactinemia [1, 20, 21].

## Diagnosis of hypoprolactinemia

The diagnostic criteria for hypoprolactinemia have not been clearly established. Different definitions and criteria have been proposed for the diagnosis of hypoprolactinemia (Tables 1 and 2). Some authors suggest to diagnose hypoprolactinemia according to basal prolactin levels, when it is either less than normal range or certain cut-off levels, while others recommend using a TRH stimulation test or antidopaminergic drug testing with metoclopramide, chlorpromazine, perphenazine, and alpha-methyl-dopa [22–26]. Although hypoprolactinemia is known to be associated with postpartum lactation failure in women, the presence of lactation after delivery does not necessarily rule out prolactin deficiency [1].

**Table 1:** Comparative analysis of basal prolactin levels for hypoprolactinemia diagnosis

Author, year	Referral for cut-off definition/ study group/Assay	Basal prolactin level for insufficiency
Mukherjee et al. 2003 [28]	Assay lower limit Patients with hypopituitarism Heterogeneous sandwich magnetic separation assay (Immuno System; Bayer Diagnostics, Newbury, Berks, UK)	Severe deficiency: < 2.4 ng/mL in at least 3 measurements Normal: ≥ 4 ng/mL
Mukherjee et al. 2006 [29]	Assay lower limit Patients with growth hormone deficiency Heterogeneous sandwich magnetic separation assay (Immuno System; Bayer Diagnostics, Newbury, Berks, UK)	Deficiency: < 1.8 ng/mL Normal: ≥ 3 ng/mL
Toledano et al. 2007 [27]	Assay lower limit Patients with hypopituitarism Immunoradiometric Immulite and Immulite 2000 assays (DPC, Los Angeles, CA)	Mild deficiency: 3–5 ng/mL Severe deficiency: < 3 ng/mL Normal: > 5 ng/mL
Corona et al. 2009 [33]	2,496 male subjects with sexual dysfuntion symptoms without hyperprolactinemia Electrochemiluminescence assay (Modular Roche, Milan, Italy)	< 5 ng/mL 1st quartile 5–7 ng/mL 2nd quartile 7–11 ng/mL 3rd quartile 11–35 ng/mL 4th quartile
Ramiandrasoa et al. 2013 [30]	Assay lower limit Patients with Sheehan’s syndrome Assay not mentioned	< 3 ng/mL
Diri et al. 2014 [31]	Patients with Sheehan’s syndrome and healthy controls Electrochemiluminescence immunoassay(Cobas; Roche Diagnostics, Mannheim, Germany)	Deficiency: < 4 ng/mL Normal: > 7.8 ng/mL
Kopczak et al. 2014 [35]	Not mentioned Patients with TBI or subarachnoid hemorrhage Chemiluminescence immunoassay	Female: < 2.8 ng/mL Males: < 2.1 ng/mL
Dias et al. 2024 [34]	65,795 adult individuals from the general population Chemiluminescence assay (Siemens ADVIA Centaur XP)	Worse metabolic outcome: < 7 ng/mL Gray zone: 9.58–12.87 ng/mL
Uzun et al. 2024 [36]	TRH stimulation test Patients with hypopituitarism and healthy controls Electrochemiluminescence immunoassay(Cobas; Roche Diagnostics, Mannheim, Germany)	Deficiency: Female: ≤ 7.1 ng/mL, Males: ≤ 5.7 ng/mL Normal: Female: ≥15.2 ng/mL, Males: ≥ 8.5 ng/mL

TRH: Thyrotropin-releasing hormone, TBI: traumatic brain injury

**Table 2:** Summary of TRH test protocols and prolactin response cut-offs used in hypoprolactinemia diagnosis

Author, year	Test details/Assay	Study population	Cut-offs or ranges of prolactin response to TRH used
Levy et al. 1978 [52]	500 μg iv TRH, not defined Radioimmunoassay	4 male patients with hemochromatosis Healthy controls: 18 men (19–42 years), 14 men (42–66 years)	Lower limit in the healthy controls: 16 ng/mL
Charbonnel et al. 1981 [43]	200 μg iv TRH, 0-15-30-60-120 min. prolactin levels Radioimmunoassay	19 male patients with hemochromatosis Healthy controls: 10 young men	Hypogonadism patients: 20.2 ± 5.1 ng/mL Normogonadism patients: 25.8 ± 4 ng/mL Healthy controls: 21.8 ± 3.25 ng/mL
Barbieri et al. 1985 [24]	200 μg iv TRH, 0-10-20-30-40-60-120 min. prolactin levels Radioimmunoassay	22 healthy controls 148 patients with no evident endocrine disease 191 patients with prolactinomas or possible prolactinomas 153 patients with pituitary diseases other than prolactinoma 162 patients with hyper or hypothyroidism 7 patients using dopamine antagonists 3 patients with other endocrine diseases	R value in healthy controls: 1.2–26 4% of normal subjects had an R value: 1–2 Median R value for control women: 7.4 Median R value for control men: 5.2
Marazuela et al. 1993 [47]	200 μg iv TRH, not defined Assay not mentioned	5 patients with Cushing’s syndrome before and after adrenalectomy	Normal: > 3-5-fold increase
Komorowski et al. 1994 [44]	200 μg iv TRH, 0-30-60–120 min. prolactin levels 10 mg oral metoclopromide only in patients Radioimmunoassay	8 healthy controls (4 women, 4 men) aged 18–50 8 euthyroid women with galactorrhea, aged 18–52	TRH test: Basal prolactin: 15.3 ± 2.3 ng/mL, Peak prolactin: 46.4 ± 8.8 ng/mL Metoclopramide test: Basal prolactin: 16.3 ± 2.6 ng/mL Peak prolactin: 107.7 ± 22.4 ng/mL
Ohbu et al. 1995 [49]	500 μg iv TRH, 0-30-60-90-120 min. prolactin levels Radioimmunoassay	37 healthy elderly men (mean age: 71.7 years) 5 healthy controls (age and gender not specified)	Insufficient response: < 30 ng/mL
Mukherjee et al. 2003 [28]	200 μg iv TRH, 0-20-60 min. prolactin levels Heterogeneous sandwich magnetic separation assay (Immuno System; Bayer Diagnostics, Newbury, Berks, UK)	Patients with hypopituitarism with undetectable basal prolactin levels	Normal: > 3-fold increase
Dokmetas et al. 2006 [59]	200 μg iv TRH, 0-30-60–90 min. prolactin levels Microparticle enzyme immunoassay (Abbott Laboratories, Abbott Park, IL, USA)	20 patients with Sheehan’s syndrome	Criteria not mentioned for deficiency Basal prolactin: 8.48 + 2.37 ng/mL 30. min: 11.73 + 3.09 ng/mL, 60. min: 12.01 + 3.21 ng/mL 90. min: 11.67 + 3.09 ng/mL
Gei-Guardia et al. 2011 [45]	200 μg iv TRH, 0-30-60 min. prolactin levels Enzyme-linked immunoassay	11 patients with Sheehan syndrome 23 age-matched healthy women	Insufficient response: < 20 ng/mL
Diri et al. 2014 [31]	200 μg iv TRH, 0-20-60 min. prolactin levels Assay not mentioned	37 patients with Sheehan’s syndrome 16 healthy controls	Insufficient response: < 2-fold increase
Triggianese et al. 2015 [48]	200 μg iv TRH, 0-15-30–60 min. prolactin levels Highly sensitive electrochemiluminescent immunoassay	Patients with 31 primary infertility and 69 recurrent spontaneous abortion 30 BMI and age matched healthy fertile women	Insufficient response: <3-5-fold increase Basal prolactin: 11.57±3.8 ng/mL Absolute increase: 62.8–44.6%, Relative increase:5.9–3.7%
Dugeroglu et al. 2018 [46]	200 μg iv TRH, 0-30-60-90-120 min. prolactin levels Assay not mentioned	32 patients with Sheehan syndrome and 40 patients with other hypopituitarism	Normal: > 30–35 ng/mL
Teramoto et al. 2023 [50]	500 μg iv TRH, 0-15-30-60-90–120 min. prolactin levels Assay not mentioned	Patients with nonfunctioning pituitary adenoma before and after surgery [70 early elderly (65–74 years) and 29 late elderly patients (≥ 75 years)]	Insufficient response: < 2-fold increase
Uzun et al. 2024 [36]	200 μg iv TRH, 0-20-60 min. prolactin levels Electrochemiluminescence immunoassay(Cobas; Roche Diagnostics, Mannheim, Germany)	48 patients with hypopituitarism 20 healthy controls	Normal: Male:> 18.6 ng/mL, Female:> 41.6 ng/mL

TRH: Thyrotropin-releasing hormone, R Value: Peak prolactin-Basal prolactin/Basal prolactin, BMI: Body mass index

### Basal prolactin levels (Table 1)

The serum prolactin reference range, while showing slight variations according to commercial assays, is generally 5–17 ng/mL in males and 5–20 ng/mL in females [27]. Toledano et al. considered serum prolactin levels between 3 and 5 ng/mL as mild and below 3 ng/mL as severe hypoprolactinemia and retrospectively evaluated 100 consecutive patients with hypopituitarism. Severe hypoprolactinemia was found in 14 patients, with the most common underlying causes being pituitary apoplexy, craniopharyngioma, and nonfunctioning pituitary adenoma, respectively. Mild prolactin deficiency was detected in 13 patients, with the most common underlying causes being idiopathic hypogonadotropic hypogonadism, pituitary apoplexy, and nonfunctioning pituitary adenoma, respectively. 71% of those with severe prolactin deficiency had 3–4 pituitary hormone deficiencies and were associated with more aggressive pituitary damage [27]. Mukherjee et al. defined hypoprolactinemia as having prolactin levels below 2.4 ng/mL on at least 3 measurements [28], which is higher than 1.8 ng/mL that they have suggested previously [29]. They identified acquired prolactin deficiency in 22 out of 369 patients with hypopituitarism [28] and demonstrated an independent association between prolactin deficiency and lower serum insulin-like growth factor-1 (IGF-1) levels in severe growth hormone (GH) deficiency patients [29]. Prolactin levels < 3 ng/mL [30], < 4 ng/mL [31, 32], < 5 ng/mL [33], and < 7 ng/mL [34] have been suggested to define hypoprolactinemia. Ramiandrasoa et al. detected prolactin deficiency (< 3 ng/mL) in 14 out of 24 patients with SS [30]. Corona et al. evaluated the role of low-to-normal prolactin levels in 2531 male subjects with sexual dysfunction without hyperprolactinemia, categorizing patients according to prolactin quartiles. Hypoprolactinemia (< 5 ng/mL) was found to be associated with anxiety, erectile dysfunction, premature ejaculation, and metabolic syndrome [33]. Dias et al. evaluated 65,795 individuals from the general population to investigate the relationship between mean prolactin levels and metabolic outcomes. They found that prolactin levels considered as hypoprolactinemia (< 7 ng/ml) were associated with worse metabolic outcomes [34]. Some authors recommended using sex-spesific cut-off levels of prolactin, 2.1 ng/mL for males and 2.8 ng/mL for females [35], and 5.7 ng/mL for males and 7.1 ng/mL for females [36]. Kopczak et al. evaluated 509 patients with TBI and subarachnoid hemorrhage for post-traumatic hypopituitarism. Prolactin deficiency (< 2.1 ng/mL for males and < 2.8 ng/mL for females) was found in only one patient (0.2%) [35]. A basal prolactin level > 7.8 ng/mL was suggested to exclude hypoprolactinemia in patients with SS [31]. So although most patients can be categorized for prolactin sufficiency and insufficiency with basal prolactin levels, there can still be a group of patients in the gray zone.

Currently, prolactin levels are generally measured using automated immunoassays; however, different assays have been used in various studies. Thus, the reference ranges for prolactin can vary depending on the assay used [37]. Additionally, the lack of standardization of prolactin assays can lead to unclear cut-off levels in the diagnosis of hypoprolactinemia.

### TRH stimulation test (Table 2)

Thyrotropin-releasing hormone, although not a major stimulant for prolactin, stimulates synthesis and secretion of prolactin in lactotroph cells. But prolactin levels were not found to be affected in cases of TRH receptor dysfunction [38]. The TRH stimulation test was introduced in 1969 with the production of synthetic TRH. Although not standardized well, it is a test used in the diagnosis of thyroid-stimulating hormone (TSH) and prolactin deficiencies. One of the problems of TRH stimulation test is difficulties in obtaining TRH ampule [39]. In healthy individuals, intravenous (IV) administration of TRH results in an immediate release of prolactin, peaking within 20–30 min, reaching 4–10 times of the basal levels, and gradually decreasing by 120 min [40, 41]. TRH stimulation test increases the diagnostic accuracy of hypopolactinemia. Diri et al. have suggested that, compared to presence of postpartum agalactia and low basal prolactin levels in the patient, TRH test is more helpful in the definitive diagnosis of hypoprolactinemia [31].

Thyrotropin-releasing hormone can be administered intramuscularly, subcutaneously, orally, rectally, and nasally, but the most standardized method is IV administration. TRH stimulation test can be used in the differential diagnosis of inappropriate TSH secretion (thyrotropinoma and thyroid hormone resistance), central hypothyroidism, hyperprolactinemia and in the diagnosis and prognosis of acromegaly [41, 42]. The accepted procedure for the TRH stimulation test is to obtain serum sample for basal prolactin between 8:00 and 10:00 a.m. in the fasting state in the morning, and repeat sampling for stimulated prolactin levels at 20 and 60 min after the administration of IV bolus of 200 μg TRH [28]. Some authors recommend assessing prolactin levels more frequently and for an extended time period such as at 15, 30, 60, and 120 min of TRH stimulation test [43, 44]. There are different suggestions for the peak prolactin levels to be used for sufficient response to the test [45–48]. In a study conducted on healthy individuals, using a TRH stimulation test with 200 μg TRH, the mean peak prolactin levels were achieved at 30 and 60. minutes. The mean prolactin levels at 30 min were between 60 and 80 ng/mL and 40–60 ng/mL, respectively [45].

Some authors, on the other hand, have recommended using 500 μg intravenous TRH, evaluating peak prolactin levels at 15, 30, 60, 90, and 120 min, and diagnosing hypoprolactinemia if the peak level is below 30 ng/mL [49] or there is less than a 2-fold increase [50]. The prolactin responses to TRH were evaluated in 7 healthy men (aged 20 to 32) on different days with 5 different doses: 6.25 μg, 25 μg, 100 μg, 400 μg, and 800 μg. Typically, the increment in prolactin level was first observed at 5 min, peak levels were achieved between 10 and 30 min, and returned to basal values by 180 min. The maximum peak prolactin response was achieved with 100 μg TRH. There were no significant differences in peak responses of prolactin between 400 μg and 800 μg of TRH. 6.25 μg of TRH lead to the minimum peak response, which did not differ from a dose of 25 μg TRH. A gender difference was also defined in prolactin responses to TRH. The prolactin responses were found to be higher in women compared to men with 400 μg of TRH [51].

A TRH stimulation test with 500 μg was conducted in 4 male hemochromatosis patients and 18 male healthy controls. In 2 patients, a sufficient prolactin response was not observed. This finding was interpreted as indicative of impaired response to hypothalamic-pituitary stimulation and decreased pituitary reserve in hemochromatosis [52]. In healthy men, IV TRH leads to a peak in prolactin levels within 15 min, and 25–100 μg of IV TRH is sufficient for the maximum prolactin response [53]. On the other hand, higher doses of TRH such as 400 μg may reduce the diagnostic power of TRH stimulation test [24].

In prolactinoma and active hyperthyroidism, the prolactin response to the TRH stimulation test is significantly blunted [24]. In contrast, subclinical or overt hypothyroidism is associated with exaggerated prolactin response to TRH which is reversed after achieving euthyroidism [54].

Age and obesity have been shown to potentially affect the peak prolactin response in the TRH stimulation test. Studies on obesity and prolactin response are conflicting, showing either normal or decreased response. Basal prolactin levels were found to be similar in 11 obese and 16 healthy women, and the prolactin responses up to 120 min after IV administration of 200 μg TRH were evaluated. The obese group showed a blunted prolactin response [55]. This decreased response has also been demonstrated in obese men [56]. This phenomenon is thought to be related to deficits in the synthesis and action of certain neurotransmitters, particularly serotonin, in obese individuals [55].

In women, the prolactin response to TRH is higher compared to men, which is thought to be estrogen-related. On the other hand, menstrual cycle phase does not seem to affect the results of TRH stimulation test [24]. Prolactin response to TRH can be influenced by drug use and various diseases, impacting both at hypothalamic and pituitary levels. Testosterone treatment generally does not affect the prolactin response, but high-dose estrogen treatment increases the response, on the other hand, oral contraceptive treatment does not induce a similar effect with high dose estrogen [41]. Pharmacological doses of glucocorticoid treatment, DAs and dopaminergic drugs can blunt the prolactin response. Discontinuation of DAs four weeks before the test would be appropriate. Somatostatin does not affect the prolactin response, while opioid receptor blockade increases it [47, 57].

Nausea, a metallic taste in the mouth, vomiting, flushing, and increased blood pressure can be seen during TRH stimulation test [42]. TRH stimulation has been reported to lead to pituitary apoplexy in seldom cases which is thought to be due to its possible vasoconstrictive effects [58]. While generally considered a safe test, the risk of apoplexy should be considered, especially in patients with large pituitary adenomas [58].

### Diagnosis of hypoprolactinemia which includes studies that performed both baseline measurements and TRH stimulation

In a recent study by Uzun et al., 48 patients with panhypopituitarism and 20 healthy controls underwent TRH stimulation testing, evaluating basal and peak prolactin responses at 20 and 60 min after IV administration of 200 μg TRH. In the healthy control group, the 5th percentile peak prolactin response to the TRH stimulation test was considered as the cut-off levels for sufficient prolactin response, separately for males and females which were 18.6 and 41.6 ng/mL, respectively. In patients with panhypopituitarism, the peak prolactin responses to TRH were significantly lower than healthy controls. Basal and peak prolactin responses to TRH stimulation were found to be positively correlated with IGF-1 levels of the patients. Among patients with panhypopituitarism, 42 (87.5%) had hypoprolactinemia according to the suggested criteria derived from healthy individuals. Other pituitary hormone levels were similar between those with sufficient and insufficient prolactin responses. According to the sufficiency of the TRH stimulation test, cut-off levels for basal prolactin were defined as follows: A basal prolactin level of ≤ 5.7 ng/mL (100% specificity and 70% sensitivity) in males and ≤ 7.11 ng/mL (100% specificity and 80% sensitivity) in females predicted insufficient responses to the TRH stimulation test. On the other hand, a basal prolactin level of ≥ 8.5 ng/ml (100% specificity and 76% sensitivity) in males and ≥ 15.2 ng/mL (96% specificity and 66% sensitivity) in females could predict obtaining sufficient prolactin responses to TRH. Therefore, we suggest to use basal prolactin levels for evaluation of hypoprolactinemia which can be diagnostic in most of the cases if properly determined cut-off levels are used [36].

Sheehan syndrome is almost always associated with hypoprolactinemia. In a previous study, hypoprolactinemia was identified in 71 (62.2%) in 114 patients with SS if the patient had a prolactin level < 4 ng/mL and 13 of these patients were able to breast-feed. A subset of patients (*n* = 31) underwent the TRH stimulation test and none of the patients with prolactin levels < 4 ng/mL showed sufficient response to the stimulation test. Among those with prolactin levels > 7.8 ng/mL, no insufficient response was observed. For the 14 patients with prolactin levels between 4 and 7.8 ng/mL, TRH stimulation test was conducted in 12 of them, and a sufficient response was found in 5 patients. A peak prolactin level of 10.8 ng/mL in the TRH stimulation test demonstrated diagnostic sensitivity of 97.2% and specificity of 94.1% for hypoprolactinemia [31].

In a study by Mukherjee et al., hypoprolactinemia was found in 22 of 369 patients with acquired hypothalamo-pituitary axis disorders, which was rarer than in SS. Diagnosis of hypoprolactinemia was confirmed with undetectable prolactin levels. Among 22 patients with low prolactin levels, 17 of them underwent TRH stimulation test, and all, except one, had a peak prolactin level below 2.35 ng/mL. Isolated prolactin deficiency was not detected in any of the patients. Majority of the hypoprolactinemic patients had a history of Cushing disease (CD), accounting for 21% of the total 62 patients with CD. The incidence of hypoprolactinemia was higher in treated CD compared to treated nonfunctioning pituitary adenomas. This situation has been considered to be associated with the extensive pituitary surgery-related damage in CD [28].

Hypoprolactinemia is seen in majority of the patients with SS, frequency depening on the diagnostic method used. A prolactin level less than 3 ng/mL, was detected in 14 (58%) of the 24 cases with SS [30]. In another study, TRH stimulation test resulted in insufficient prolactin responses in all 20 cases with SS which was accompanied by GH deficiency and the authors suggested that in patients with severe postpartum hemorrhage, the TRH stimulation test is the most sensitive test for the diagnosis of hypoprolactinemia [59].

Although there is no clearly determined cut-off levels of prolactin response to be used during TRH stimulation test, generally an increase of prolactin less than 2 times of the basal level is considered as an insufficient response. However, we have recently shown that doubling of prolactin may lead to inaccurate results in some patients and it would be better to use peak prolactin responses to TRH [36]. A peak prolactin response of 18.6 ng/mL for males and 41.6 ng/mL for females to TRH were considered to be sufficient [36]. On the other hand, TRH stimulation is not a sine qua non for the diagnosis of hypoprolactinemia. Taking the limited availability of TRH into account, basal prolactin levels can be used in the first step and prevent unnecessary use of TRH stimulation test in most of the patients with hypopituitarism. Factors other than gender such as age, body mass index, medication use, metabolic parameters and accompanying comorbidities can also influence the evaluation of the test. The diagnosis may become more important if the effects of hypoprolactinemia is considered to be important and replacement therapy comes to the fore.

Most of the diagnostic data on prolactin deficiency are based on earlier studies, however, the increasing importance of the subject has led to gain popularity of evaluation of prolactin reserve. There is a need for further studies with larger populations and more standardized assays and reference ranges, involving both basal prolactin levels and TRH stimulation tests across different age, gender, and BMI groups, in both healthy individuals and patients with pituitary gland dysfunction, from a wider range of centers. Additionally, future studies can be conducted to evaluate the utility of different stimuli such as metoclopramide in assessing prolactin reserve.

## Conclusions

Hypoprolactinemia serves as a marker for extensive pituitary gland damage and dysfunction. Low/undetectable serum prolactin levels and the absence of a sufficient prolactin peak in the TRH stimulation test are considered as diagnostic. Gender seems to be important for both basal prolactin levels and TRH stimulation test response. A minimum basal prolactin level of 5 ng/mL for males, 7 ng/mL for females and a minimum peak prolactin response to TRH of 18 ng/mL for males and 41 ng/mL for females seem to be acceptable as a normal. However, we still need larger population studies in different age groups and sexes for normal basal prolactin levels and prolactin responses to TRH stimulation test.

## Data Availability

No datasets were generated or analysed during the current study.
